# Automated Cobb Angle Measurement for Adolescent Idiopathic Scoliosis Using Convolutional Neural Network

**DOI:** 10.3390/diagnostics12020396

**Published:** 2022-02-03

**Authors:** Wahyu Caesarendra, Wahyu Rahmaniar, John Mathew, Ady Thien

**Affiliations:** 1 Manufacturing Systems Engineering, Faculty of Integrated Technologies, Universiti Brunei Darussalam, Jalan Tungku Link, Gadong BE1410, Brunei; 2 Department of Electronic Engineering, National Taipei University of Technology, Taipei 10608, Taiwan; wahyu.rahmaniar@gmail.com; 3 Department of Neurosurgery, Brunei Neuroscience, Stroke and Rehabilitation Centre, Pantai Jerudong Specialist Centre, Jerudong BG3122, Brunei; dr.johnmathew@pjscbrunei.com

**Keywords:** convolutional neural network (CNN), deep learning, scoliosis, spine classification, vertebrae

## Abstract

The Cobb angle measurement of the scoliotic spine is prone to inter- and intra-observer variations in the clinical setting. This paper proposes a deep learning architecture for detecting spine vertebrae from X-ray images to evaluate the Cobb angle automatically. The public AASCE MICCAI 2019 anterior-posterior X-ray image dataset and local images were used to train and test the proposed convolutional neural network architecture. Sixty-eight landmark features of the spine were detected from the input image to obtain seventeen vertebrae on the spine. The vertebrae locations obtained were processed to automatically measure the Cobb angle. The proposed method can measure the Cobb angle with accuracies up to 93.6% and has excellent reliability compared to clinicians’ measurement (intraclass correlation coefficient > 0.95). The proposed deep learning architecture may be used as a tool to augment Cobb angle measurement in X-ray images of patients with adolescent idiopathic scoliosis in a real-world clinical setting.

## 1. Introduction

Scoliosis is a structural abnormality in which the spine curves from side to side and rotates. Children aged 10 to 17 years old who present with scoliosis of unknown cause are categorized as having Adolescent Idiopathic Scoliosis (AIS) [1]. Patients with mild deformities are usually asymptomatic. However, if the curvature progresses during the growth spurt, discomfort, pain, and symptoms related to abnormal chest wall growth and difference in shoulder height can lead to decreased quality of life [2]. AIS can cause respiratory symptoms, such as shortness of breath when the curvature exceeds 50°, and patients are at a high risk of significant lung function abnormalities if the curvature is more than 100° [3].

The Cobb measuring method is the gold standard used in quantifying the scoliotic curve. The Cobb Angle (CA) is measured from the most tilted vertebra (end vertebra) above and below the apex (most laterally placed vertebra) of the curve on radiographs taken either in the anterior-posterior or the posterior-anterior view on the coronal plane [4]. In general, the “manual” procedure requires lines to be drawn onto a hardcopy of radiographic films, and the angle between the two lines is measured with a protractor. Therefore, measuring the CA can be time-consuming and is also prone to inter-observer and intra-observer variations. Reported accuracies of measuring CA vary from 2° to 11° [5,6,7], with measurements differing up to 5° even with the same end vertebrae selected [2,8].

Semi-automatic assessments of CA have been made possible with the advent of the digitalization of computerized radiography. The Picture Archiving and Communications System (PACS) enables a built-in function so users can digitally draw lines for the required vertebra, and the system will automatically measure the CA. This method has been proven reliable and has less variation than the manual method [7,9]. However, this method is still dependent on the user selecting the appropriate end vertebrae manually.

With the advancement of computer vision technology [10], such as machine learning [11,12] and deep learning methods [13,14,15], a fully automatic process to measure the CA quickly and precisely can potentially overcome the shortcomings mentioned above. Digitalized X-ray images, processed through computerized learning, have previously been used to screen, diagnose, and classify severity of scoliosis [12]. Further development in deep learning has also led to an increase in different measuring and assessing CA [13,14,15,16]. Automatic detection of the spine anatomy in X-ray images to select the appropriate end vertebrae is an essential stage for CA measurement. Studies on the vertebrae and spine detection based on machine learning [11,12] and deep learning methods [13,14,15,16] have been previously reported. However, automatic detection of the vertebrae in X-ray images can be difficult due to overlapping rib and pelvic shadows, as well as contrast differences between the different vertebrae levels. Convolutional neural network (CNN) architecture has been previously shown to help overcome this problem [17].

In this paper, we propose a CNN method for the automatic detection of spinal vertebrae and the measurement of CA in X-ray images. The proposed Automated CA Measurement Method (ACAMM) is assessed for accuracy and reliability compared to clinicians’ CA measurements.

## 2. Materials and Methods

This study used two datasets which were comprised of open-source and local data. CNN was used for automated spinal detection and CA measurement. A total of 551 X-ray images were evaluated; 481 and 70 X-ray images were used for training and testing stages, respectively. For ease of calculation, the collected data were divided into (1) CA < 10°; (2) CA 10° to 25°; (3) CA > 25° to 40°; and (4) CA > 40°.

A detailed description of the datasets and the methods are presented in the following subsections.

### 2.1. Opensource Datasets

**Figure 1:**
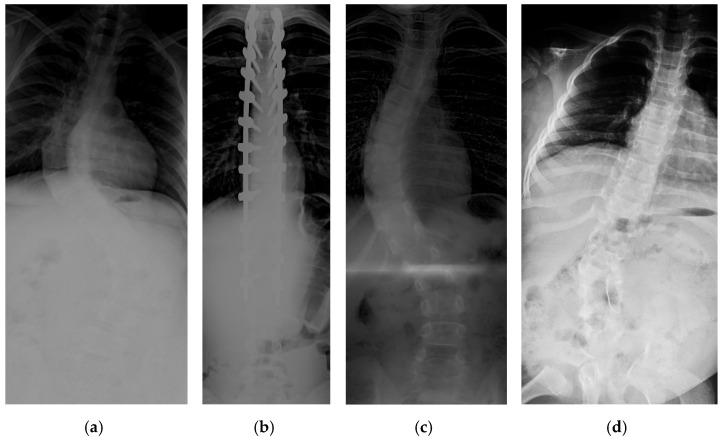
Examples of some challenging image conditions: (**a**) Image with noise; (**b**) high contrast image; (**c**) low light image; (**d**) spine with high CA.

The collection and labeling of spinal images were performed by the public AASCE MICCAI 2019 anterior-posterior X-ray images dataset [18]. The input images vary in size from 359 × 973 to 1427 × 3755. Some challenging images can be handled due to our large number of training image conditions, which include images with different noise, contrast, lighting conditions, and spines with high CA, as shown in Figure 1. Each image contains 17 vertebrae from the thoracic (upper spine) and lumbar (lower spine) regions. The image input resolution is set to 1024 × 512 for the algorithm development. Each vertebra is located by four corner landmarks. The ground-truth of the 68 landmarks or points in each image is provided by the dataset.

### 2.2. Local Datasets

**Figure 2:**
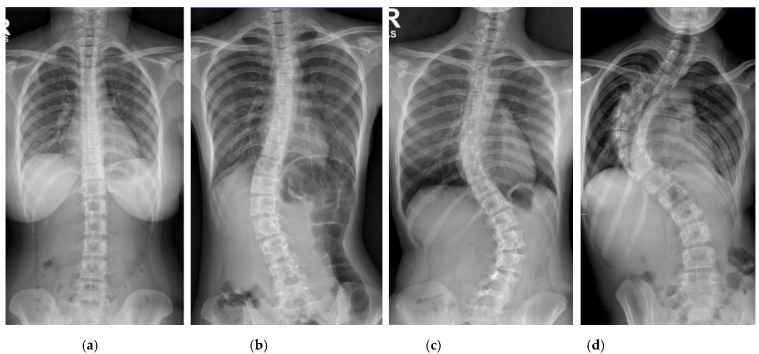
Examples of standard X-ray images exported from the PACS: (**a**) CA < 10°; (**b**) CA 10° to 25°; (**c**) CA > 25° to 40°; (**d**) CA > 40°.

Patients with AIS who attended the scoliosis clinic at Pantai Jerudong Specialist Centre from 1 November 2018 to 4 September 2020 were identified from the institution’s scoliosis database. These patients had standard standing anterior-posterior X-rays showing cervical vertebra level 7 to the femoral heads and the entire rib cage from right to left as part of routine clinical management. The X-ray images were retrieved from the institution PACS, anonymized, and exported as a JPEG format image, as shown in Figure 2. The CA for each X-ray image was measured by (1) two neurosurgeons (Observer 1 specializes in scoliosis; Observer 2 does not), who were blinded to each other and the ACAMM, using the in-built function in the PACS and (2) the ACAMM.

### 2.3. Proposed Methods

**Figure 3:**
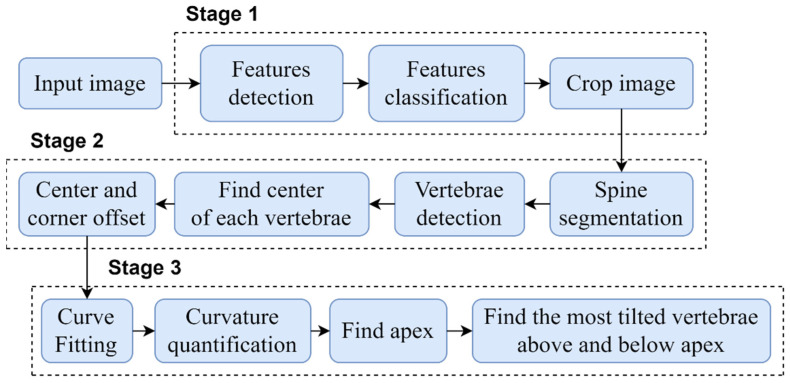
A block diagram of the proposed method.

The proposed automated vertebrae detection and CA measurement comprises of sequential stages presented in detail in Figure 3.

In order to automate the CA measurement, this study was performed in three main stages: (1) development of an algorithm to automatically crop and standardize the X-ray images dimension; (2) development of vertebrae detection based on the local X-ray images using CNN which improved the previous method described [19,20]; and (3) development of an algorithm to identify the apex vertebra and superior and inferior end vertebrae to measure the CA. The following subsections provide a detail description of the three main stages.

#### 2.3.1. Stage 1: Pre-Processing of the X-ray Images Size

**Figure 4:**
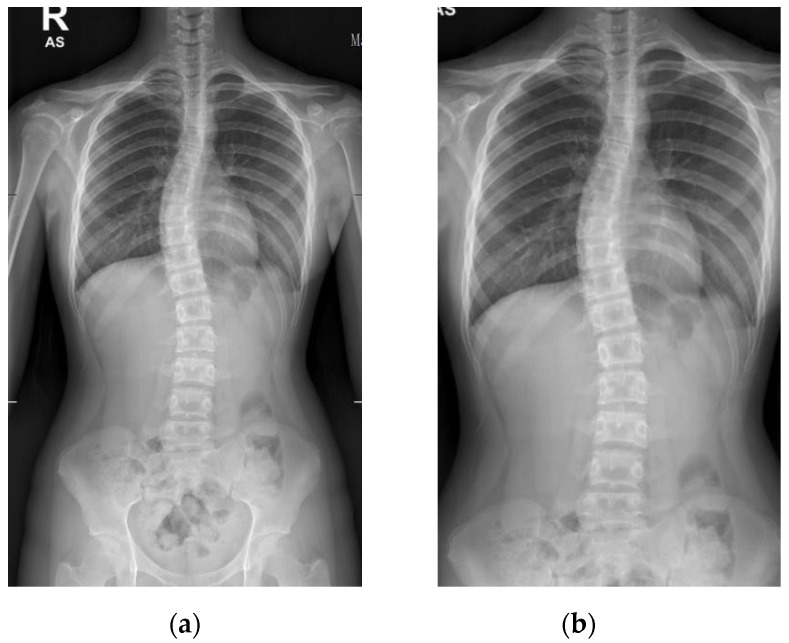
Pre-processing of the X-ray image: (**a**) original; (**b**) post-processing.

Local Binary Patterns (LBP) and cascade classifier [21], a type of visual descriptor used for classification in computer vision, was used to standardize the X-ray images and automatically crop the image between the cervical and sacrum. An example of the process result is shown in Figure 4.

The LBP code label histogram contains information about the pixel level distribution for edges and other local features in the image. This feature was chosen because it uses a derivative pattern to obtain direction from a binary gradient, making it suitable for obtaining information from X-ray images that have a gray-scale color. Following this, a cascade classifier is used to select features from the X-ray image that are used to define the body area of the object. This body region is selected and used as a positive input for training the cascade classifier. The limits of the boundaries were from cervical vertebrae level-7 to the sacrum at the lumbar-sacral junction, and the right and left outermost parts of the body. The negative image which is separate from the body region of interest is also obtained. The pixel information which contains about body region can be labeled as

(1) $$LBP_{s,r}(N_{c})=\sum_{s=0}^{s-1}g\left(z\right)2^{s}$$

where $z=N_{s}-N_{c}$, *s* is the number of sampling points in a small circular neighborhood with *r* = 1, $N_{s}$ is the neighborhood pixels in each *s*, $N_{c}$ is the neighborhood center pixel, and the binary threshold function *g*(*x*) can be defined as

(2) $$g(z)=\left\{ \begin{array}{ll} 1,\; & z\geq 0\\ 0,\; & z<0 \end{array}\right.$$

This body region is then detected by the sliding window method and each area traversed by each sub-window is labeled as positive or negative, can be represented as

(3) $$L(X)=\left\{ \begin{array}{ll} 1, & \mathrm{if}\;\sum_{i=1}^{I}\left[\alpha_{i}(X)\times \log\left(\frac{1}{\beta_{i}}\right)\right]\geq \frac{1}{2}\sum_{i=1}^{I}\log\left(\frac{1}{\beta_{i}}\right)\\ 0, & \mathrm{otherwise} \end{array}\right.$$

where *X* is a set of the training images, *α* is the weight vector, *β* is the weighting parameter which computed from the error associated with the classifier, *i* = 1, 2, …, *I* is the iteration number of the training. If the classifier detects the label as positive, the detection is passed to the next stage. Continuation of the classifier until the final stage will enable the production of a body region to be used as an input image in the next stage.

#### 2.3.2. Stage 2: Vertebrae Detection on the Local X-ray Images Using CNN

The deep learning architecture was based on CNN for automated spine segmentation to select the vertebrae accurately and determine the center and corner offset of each vertebra based on a method described previously [19]. Modifications in the pre-processing method and parameter selection were performed according to local datasets. In addition, we improved upon the previous method to enable the automatic CA measurement. 

**Figure 5:**
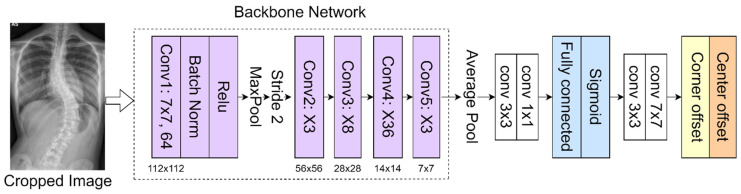
Vertebrae detection network.

In the proposed method, the 152-layer ResNet [22] is used as a backbone network to classify 68 landmarks to obtain the corner offset of spine. This CNN [23,24,25] consists of several convolutional layers that learn the local features of the images and generate the classifications. A bottleneck block with 4-layer extension and 152 layers was built using more 3-layer blocks for higher accuracy [26,27]. Despite having increased layers, this backbone has a lower complexity than the ResNet-50 used in the previous method [19]. This feature map is then classified into a fully connected layer with a sigmoid function to get a better feature intensity. The proposed network as presented in Figure 5 includes pooling layers (average pool and maximum pool), feature maps classification (fully connected layer and sigmoid function), and corner offset. The model is initialized from the pre-trained weights on ImageNet. The network was trained to a learning rate of 0.0001 using the Adam optimizer; the batch and epoch sizes were set as 2 and 150, respectively.

The parameters to obtain the classification were optimized using the focal loss [19] as follows:

(4) $$Loss=-\frac{1}{M}\sum_{m=1}^{M}\left\{ \begin{array}{ll} {\left(1-\rho_{m}\right)}^{2}\log\rho_{m} & ,\;\tau_{m}=1\\ {\left(1-\tau_{m}\right)}^{4}{\left(\rho_{m}\right)}^{2}\log(1-\rho_{m}) & ,\;\mathrm{otherwise} \end{array}\right.$$

where *m* = 1, 2, …, *M* is the index of each feature maps’ position, $\rho_{m}$ and $\tau_{m}$ are the prediction and ground-truth value, respectively. 

**Figure 6:**
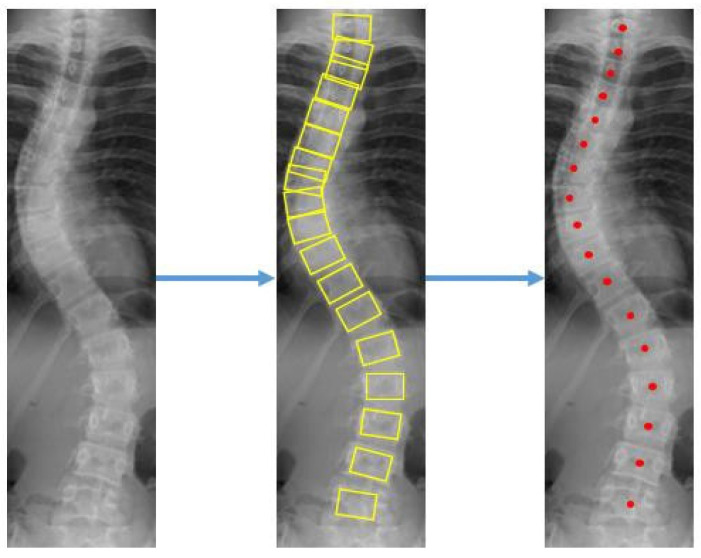
Progressive results of the vertebrae detection to get the location of 4 corners and a center.

The center offset and corner offset maps using convolutional layers for landmark localization were constructed. Since the output of the feature map on the network is downsized, the center offset and corner offset are mapped to a new location which is then trained with L1 loss. Detection bounding boxes were displayed on each vertebra after applying the object detection step on the X-ray images. The coordinates for the corners and center of each bounding box were found as presented in Figure 6.

Errors in the detection of landmarks in the vertebrae are evaluated by

(5) $$\varepsilon =\frac{1}{T}\sum_{t=1}^{T}{\left\|\left(d_{x,t},d_{y,t}\right)-\left(p_{x,t},p_{y,t}\right)\right\|}_{2}$$

where *d* and *p* are the locations (*x*,*y*) of the detected and ground-truth landmarks, respectively, *t* = 1, 2, …, *T* is the total number of the detected landmarks.

#### 2.3.3. Stage 3: Cobb Angle (CA) Measurement

**Figure 7:**
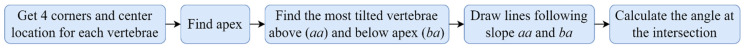
Steps to measure the CA.

Curve fitting was used to select the appropriate boxes. Boxes with a prediction score of more than 0.5 were extracted. From the location of the detected boxes, the center point of each vertebra is found to remove some outliers based on the anatomy of the spine, where the adjacent vertebrae should not be far apart from each other. If the *x*-axis center of the detected bounding box is more than half the width of the box from the *x*-axis center of its two closest neighbors (top and bottom), the box is rejected as an outlier. Otherwise, the position of the box is reconsidered based on the position of the nearest boxes. The steps to measure the CA is presented in Figure 7.

**Figure 8:**
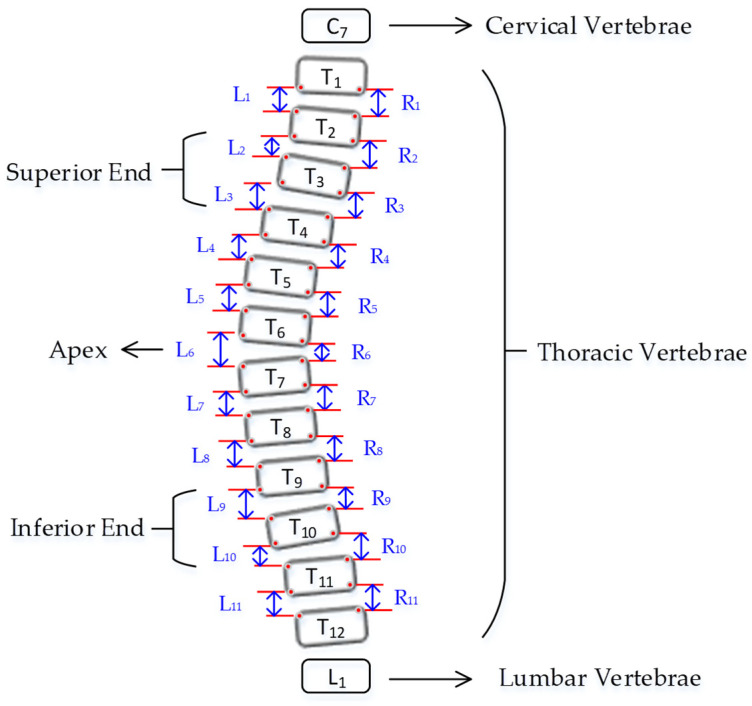
Vertebrae anatomy for the illustration of finding apex, superior end, and inferior end- vertebrae.

**Figure 9:**
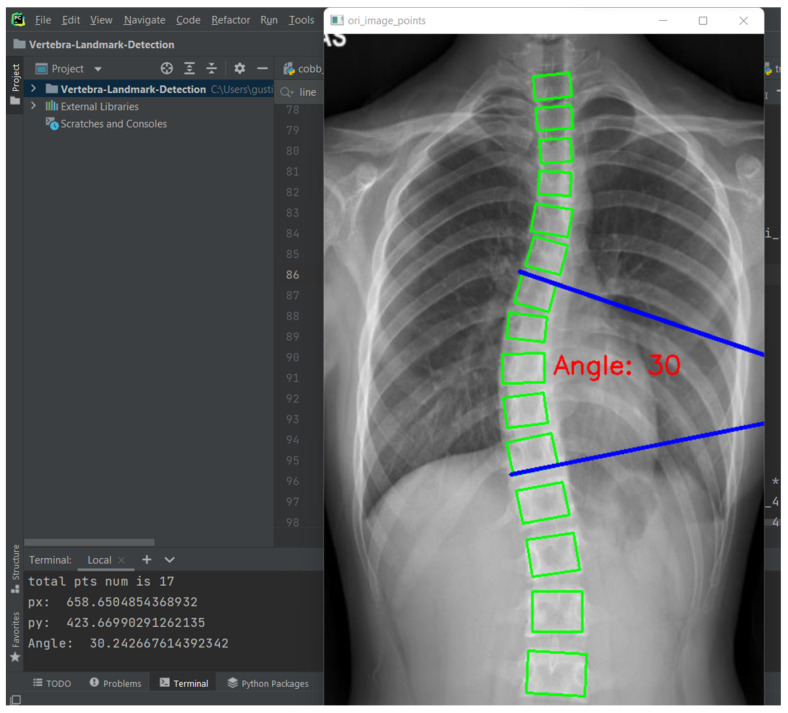
Automated vertebrae detection and CA measurement.

Curvature quantification, the depth of the curve at the found position of the corner box, was obtained (Figure 8). For each of the two vertebrae, the distances between the bottom-left point of the upper box and the top-left of the lower box (L1-L11), and the bottom-right point of the upper box and the top-right of the lower box (R1-R11) were calculated. The apex of the spine curvature is found as the deepest part of the curve. For each box above the apex, the slope of each vertebrae is measured based on the position between top-left and top-right to detect the most-tilted vertebrae above the apex. For each box below the apex, the slope of each vertebra is measured based on the position between bottom-left and bottom-right to detect most-tilted vertebra below the apex. The location of the superior end vertebra and inferior end vertebra is identified as the most tilted vertebra above apex (*aa*) and below apex (*ba*), respectively. CA measured as the angle of the intersection between two lines from *aa* to *ba*. Figure 9 shows an example of a measurement performed by the ACAMM.

### 2.4. Statistical Analysis

All statistical analyses were performed with SPSS version 20 (IBM Corporation, Armonk, New York, United States of America). The chi-squared test and the Mann-Whitney *U* test were performed for nominal and non-normally distributed variables, respectively. Using Observer 1 as the reference, median percentage accuracy (Interquartile Range (IQR)), median CA measurement differences (IQR), and proportion of CA measurements within ±5° comparing the ACAMM to Observer 2 was calculated. The percentage accuracy was calculated based on the following formula:

(6) $$\mathrm{Percentage}\;\mathrm{accuracy}\;(\%)=100-100\left(\left|\frac{\bar{C}\;-\overset{´}{C}}{\bar{C}}\right|\right)$$

where $\bar{C}$ is the Observer 1 CA measurement and $\overset{´}{C}$ is ACAMM (or Observer 2) CA measurement.

The reliability of the ACAMM using our proposed CNN was assessed by Intraclass Correlation Coefficient (ICC) and Pearson Correlation Coefficient (PCC). Generally, the ICC reliability values are rated as poor (<0.50), fair (0.50 to 0.75), good (>0.75 to 0.90), or excellent (>0.90). The significance level for the study was set at *p* < 0.05.

## 3. Results

### 3.1. Vertebrae Detection Results

The datasets were trained on the RTX2060 GPU with Intel Core-i7 processor. The proposed architecture using CNN accurately detected the location of each of the 17 vertebrae in the spine X-ray. In addition to this, the bounding box was evaluated to be sufficient in its accordance with the vertebra positions. Its performance was accurate to provide the information needed to detect the superior and inferior end vertebrae, enabling the CA to be evaluated correctly.

**Figure A1:**
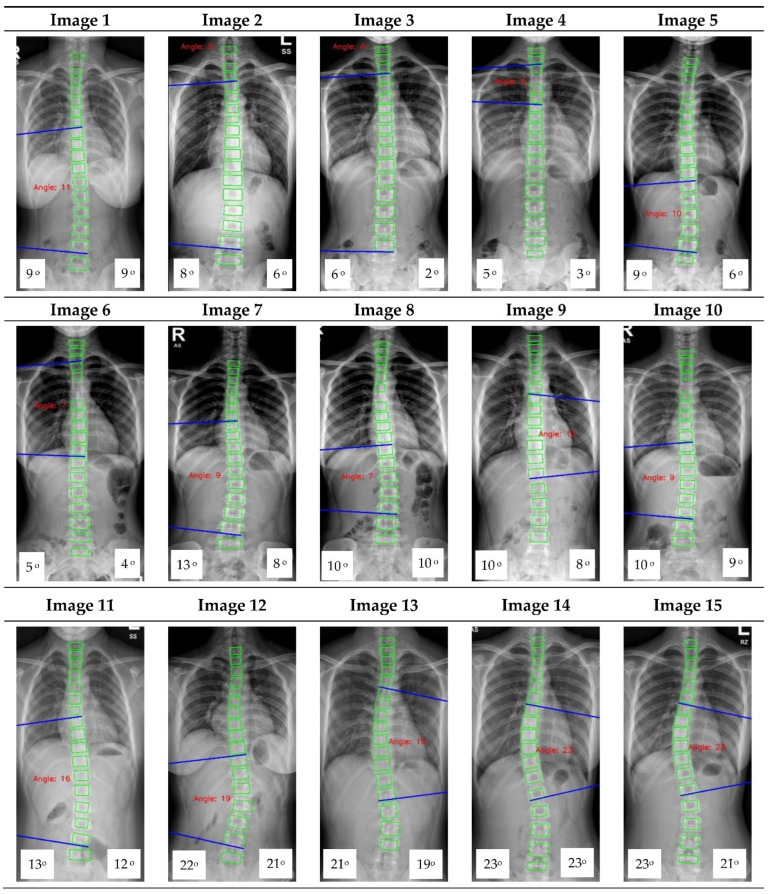

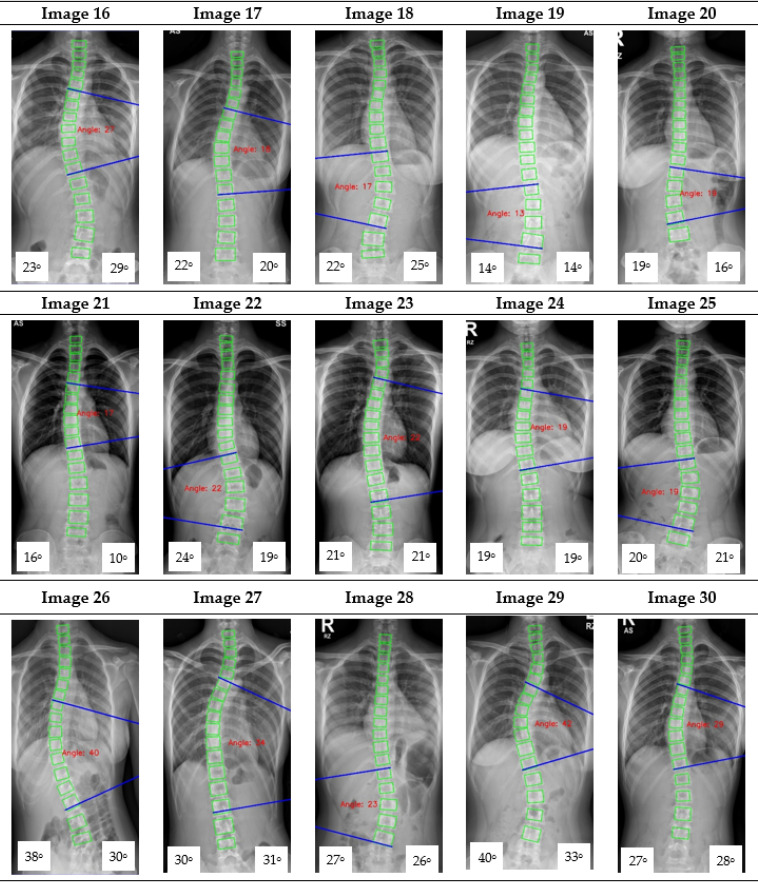

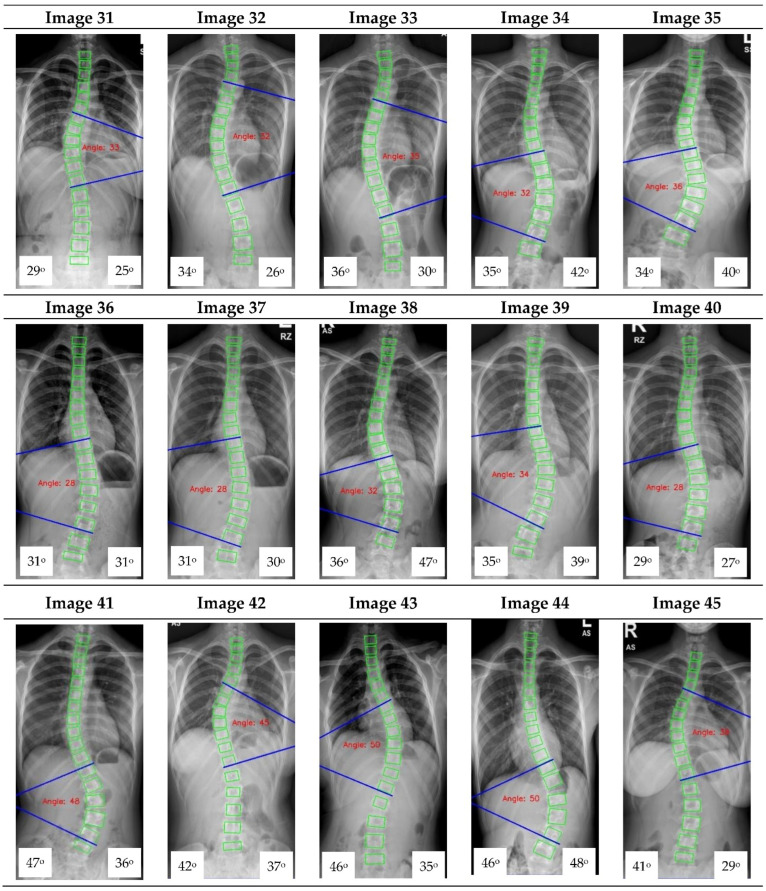

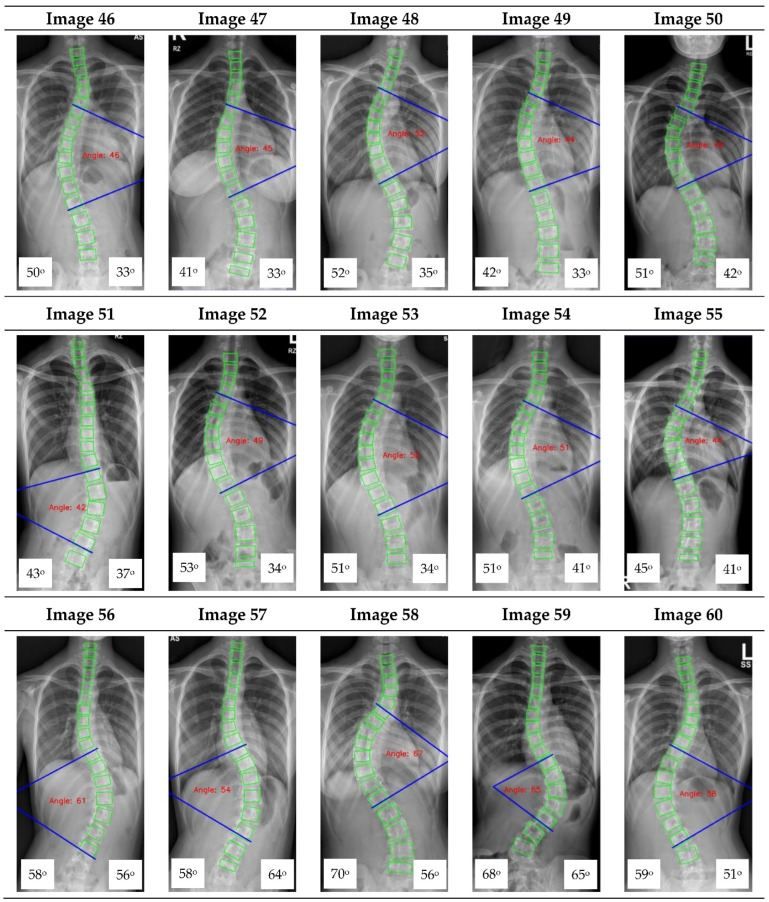

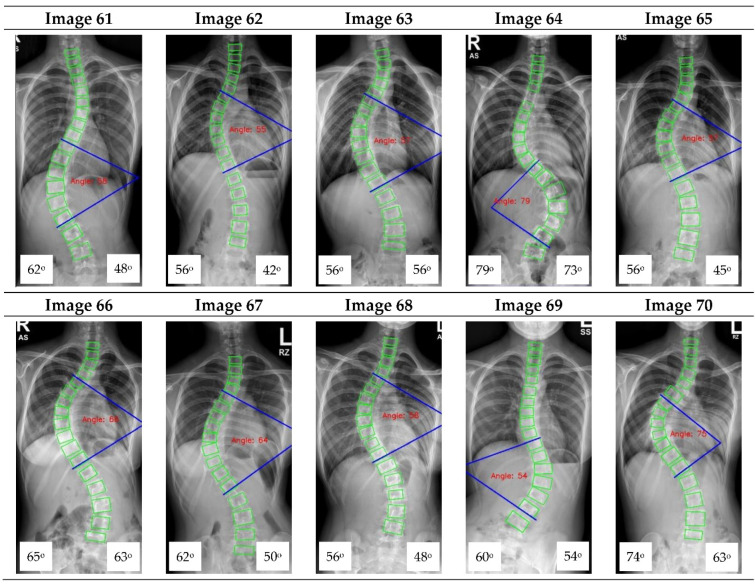
The red font inside the X-ray image is a CNN CA measurement. Observer 1 and 2 evaluation is presented on the bottom left and right, respectively.

The detection results also showed that the proposed architecture could be used to identify the vertebrae in X-ray images of different contrast and lighting conditions (see Appendix A, Figure A1). Our test on several images with poor contrast and lighting conditions yielded good results. Importantly, CA measurements and curve classification were able to be accurately accomplished even when the detection process failed to identify one or two vertebrae due to our curve fitting and quantification method. Previous studies using CNN [14,16] focused on vertebrae detection and measurement of CA under certain conditions. The method we proposed was able to measure CA from normal to severely scoliotic spine (up to 79°). This is a key part of the algorithm as X-ray images may come in different contrast and lighting qualities in the clinical setting, depending on the severity of the curve as well as the patient’s body habitus.

### 3.2. Evaluation of Automated CA Measurement Results and Ground Truth

The results of the ACAMM were compared with the results measured by two Neurosurgeons (Observer 1 specializes in scoliosis; Observer 2 does not) as shown in Table 1, Table 2, Table 3 and Table 4. Using Observer 1 as the reference, the ACAMM showed better median accuracy (93.6% [89.7% to 97.7%] vs. 85.9% [78.3% to 95.5%], *p* < 0.001), smaller median CA measurement differences (0° [−3.0° to 2.0°] vs.−3.0° [−8.0° to 0°], *p* < 0.001) and higher percentage of CA measurements within ± 5° (98.6% [69/70] vs. 54.3% [38/70], *p* = 0.001), compared to Observer 2 (Table 5). 

Overall, the ACAMM was highly matched to the CA assessment performed by the two observers (Table 6). ICC for the ACAMM compared to Observer 1 and Observer 2 was 0.995 and 0.954, respectively. The PCC also showed high correlation between the ACAMM and Observer 1 (0.991, *p* < 0.001) and Observer 2 (0.931, *p* < 0.001). 

## 4. Discussions

Our proposed method of automatic assessment of CA on spine X-ray images used ResNet-152 as a backbone to improve the performance accuracy. We used feature maps classification with a sigmoid function as network depth has previously been shown to be beneficial in classification accuracy. However, its performance can become saturated with a resultant rapid decrease in performance as the network gains greater depth. This issue can be corrected by the ResNet framework, where a shortcut connection is added for every three convolution layers across the deep network. These shortcut connections performed identity mapping without additional parameters, which can increase computational complexity. This simplification of network optimization during the training process enabled ResNet to achieve a higher accuracy from deeper networks when performing image segmentation tasks. Curve fitting and quantification were used to handle errors in vertebrae detection so that accurate CA measurements could still be obtained.

Reproducibility remains a common problem in CA measurements due to the high degree of intra-observer and inter-observer variabilities. An objective, reliable method to determine CA is crucial, as this measurement is used to determine and guide clinical decisions regarding diagnosis, curve progression, and management, including surgical options. Our ACAMM showed good CA measurement accuracies (93.6%) when assessed against a neurosurgeon with expertise in scoliosis. Importantly, all except for one (98.6%) of the automated measurements were within ±5° in CA measurements of the expert neurosurgeon. Generally, this variation is within the accepted threshold when measuring CA in the clinical setting [5,8]. This was in contrast to the second observer, where only 54.3% of the CA measurements were within ±5°. Furthermore, the reliability of our proposed method to measure CA was excellent (ICC > 0.95, PCC > 0.93). The results, which are highly matched with the assessment performed by the two neurosurgeons, indicate that this CNN method has a high potential for its use in the real-world setting. 

There are limitations to this study. The results shown were performed in a non-clinical setting. Therefore, further tests of reliability and accuracy in the clinical setting with real-time comparisons with the clinicians’ CA measurement, including an increase in the testing samples and observers, is warranted. Secondly, the current method is only able to measure a single major curvature in the spine and cannot detect other minor curves in the same X-rays, which may be clinically relevant. Future work to enable the ability of the algorithm to detect all the curves in the scoliotic spine X-ray will be explored.

## 5. Conclusions

We developed a pre-processing method and a deep learning architecture using a convolutional neural network for spine segmentation and vertebrae detection to automatically measure Cobb angle in adolescent idiopathic scoliosis. The vertebrae detection network uses ResNet-152 as the backbone, feature maps classification, and corner offset compensation to improve the performance accuracy. The proposed method demonstrates good measurement accuracies when compared against an expert in scoliosis and has an excellent reliability rating, indicating it is a promising method for automatic measurement of Cobb angle in a real-world setting. 

## Figures and Tables

**Table 1:** X-ray images evaluation for Cobb angle <10°.

Image	Observer 1 Reference CA Measurement (°)	Observer 2 CA Measurement (°)	Observer 2–Observer 1 CA Difference (°)	CA Difference within ±5°	Observer 2 Accuracy (%)	ACAMM (°)	ACAMM–Observer 1 CA Difference (°)	CA Difference within ±5°	ACAMM Accuracy (%)
1	9	9	0	Yes	100.0	11	+2	Yes	77.8
2	8	6	−2	Yes	75.0	8	0	Yes	100.0
3	6	2	−4	Yes	33.3	4	−2	Yes	66.7
4	5	3	−2	Yes	100.0	6	+1	Yes	40.0
5	9	6	−3	Yes	66.7	10	+1	Yes	88.9
6	5	4	−1	Yes	80.0	7	+2	Yes	60.0

Note: Cobb Angle (CA); Automated Cobb Angle Measurement Method (ACAMM).

**Table 2:** X-ray images evaluation for Cobb angle 10° to 25°.

Image	Observer 1 Reference CA Measurement (°)	Observer 2 CA Measurement (°)	Observer 2–Observer 1 CA Difference (°)	CA Difference within ±5°	Observer 2 Accuracy (%)	ACAMM (°)	ACAMM–Observer 1 CA Difference (°)	CA Difference within ±5°	ACAMM Accuracy (%)
7	13	8	−5	Yes	61.5	9	−4	Yes	69.2
8	10	10	0	Yes	100.0	7	−3	Yes	70.0
9	10	8	−2	Yes	80.0	12	+2	Yes	80.0
10	10	9	−1	Yes	90.0	9	−1	Yes	90.0
11	13	12	−1	Yes	92.3	16	+3	Yes	76.9
12	22	21	−1	Yes	95.5	19	−3	Yes	86.4
13	21	19	−2	No	90.5	19	−2	Yes	90.5
14	23	23	0	Yes	100.0	23	0	Yes	100.0
15	23	21	−2	Yes	91.3	23	0	Yes	100.0
16	23	29	+6	No	73.9	27	+4	Yes	82.6
17	22	20	−2	Yes	90.9	18	−4	Yes	81.8
18	22	25	+3	Yes	86.4	17	−5	Yes	77.3
19	14	14	0	Yes	100.0	13	−1	Yes	92.9
20	19	16	−3	Yes	84.2	19	0	Yes	100.0
21	16	10	−6	No	62.5	17	+1	Yes	93.8
22	24	19	−5	Yes	79.2	22	−2	Yes	91.7
23	21	21	0	Yes	100.0	22	+1	Yes	95.2
24	19	19	0	Yes	100.0	19	0	Yes	100.0
25	20	21	+1	Yes	95.0	19	−1	Yes	95.0

Note: Cobb Angle (CA); Automated Cobb Angle Measurement Method (ACAMM).

**Table 3:** X-ray images evaluation for Cobb angle >25° to 40°.

Image	Observer 1 Reference CA Measurement (°)	Observer 2 CA Measurement (°)	Observer 2–Observer 1 CA Difference (°)	CA Difference within ±5°	Observer 2 Accuracy (%)	ACAMM (°)	ACAMM–Observer 1 CA Difference (°)	CA Difference within ±5°	ACAMM Accuracy (%)
26	38	30	−8	No	78.9	40	+2	Yes	94.7
27	30	31	+1	Yes	96.7	34	+4	Yes	86.7
28	27	26	−1	Yes	96.3	23	−4	Yes	85.2
29	40	33	−7	No	82.5	42	+2	Yes	95.0
30	27	28	+1	Yes	96.3	29	+2	Yes	92.6
31	29	25	−4	Yes	86.2	33	+4	Yes	86.2
32	34	26	−8	No	76.5	32	−2	Yes	94.1
33	36	30	−6	No	83.3	35	−1	Yes	97.2
34	35	42	+7	No	80.0	32	−3	Yes	91.4
35	34	40	+6	No	82.4	36	+2	Yes	94.1
36	31	31	0	Yes	100.0	28	−3	Yes	90.3
37	31	30	−1	Yes	96.8	28	−3	Yes	90.3
38	36	47	+11	No	69.4	32	−4	Yes	88.9
39	35	39	+4	Yes	88.6	34	−1	Yes	97.1
40	29	27	−2	Yes	93.1	28	−1	Yes	96.6

Note: Cobb Angle (CA); Automated Cobb Angle Measurement Method (ACAMM).

**Table 4:** X-ray images evaluation for Cobb angle > 40°.

Image	Observer 1 Reference CA Measurement (°)	Observer 2 CA Measurement (°)	Observer 2–Observer 1 CA Difference (°)	CA Difference within ±5°	Observer 2 Accuracy (%)	ACAMM (°)	ACAMM–Observer 1 CA Difference (°)	CA Difference within ±5°	ACAMM Accuracy (%)
41	47	36	−11	No	76.6	48	1	Yes	97.9
42	42	37	−5	Yes	88.1	45	3	Yes	92.9
43	46	35	−11	No	76.1	50	4	Yes	91.3
44	46	48	2	Yes	95.7	50	4	Yes	91.3
45	41	29	−12	No	70.7	38	−3	Yes	92.7
46	50	33	−17	No	66.0	46	−4	Yes	92.0
47	41	33	−8	No	80.5	45	4	Yes	90.2
48	52	35	−17	No	67.3	52	0	Yes	100.0
49	42	33	−9	No	78.6	44	2	Yes	95.2
50	51	42	−9	No	82.4	49	−2	Yes	96.1
51	43	37	−6	No	86.0	42	−1	Yes	97.7
52	53	34	−19	No	64.2	49	−4	Yes	92.5
53	51	34	−17	No	66.7	52	1	Yes	98.0
54	51	41	−10	No	80.4	51	0	Yes	100.0
55	45	41	−4	Yes	91.1	44	−1	Yes	97.8
56	58	56	−2	Yes	96.6	61	3	Yes	94.8
57	58	64	6	No	89.7	54	−4	Yes	93.1
58	70	56	−14	No	80.0	67	−3	Yes	95.7
59	68	65	−3	Yes	95.6	65	−3	Yes	95.6
60	59	51	−8	No	86.4	58	−1	Yes	98.3
61	62	48	−14	No	77.4	58	−4	Yes	93.5
62	56	42	−14	No	75.0	55	−1	Yes	98.2
63	56	56	0	Yes	100.0	57	1	Yes	98.2
64	79	73	−6	No	92.4	79	0	Yes	100.0
65	56	45	−11	No	80.4	57	1	Yes	98.2
66	65	63	−2	Yes	96.9	66	1	Yes	98.5
67	62	50	−12	No	80.6	64	2	Yes	96.8
68	56	48	−8	No	85.7	58	2	Yes	96.4
69	60	54	−6	No	90.0	54	−6	No	90.0
70	74	63	−11	No	85.1	75	1	Yes	98.6

Note: Cobb Angle (CA); Automated Cobb Angle Measurement Method (ACAMM).

**Table 5:** Comparison between automated Cobb angle measurement method and Observer 2.

	ACAMM vs. Observer 1	Observer 2 vs. Observer 1	*p*
**Overall**			
Median accuracy, % (IQR)	93.6 (89.7 to 97.7)	85.9 (78.3 to 95.5)	<0.001
Median CA difference, ° (IQR)	0 (−3.0 to 2.0)	−3.0 (−8.0 to 0)	<0.001
CA difference within ±5°, n (%)	69/70 (98.6)	38/70 (54.3)	<0.001
**CA < 10°**			
Median accuracy, % (IQR)	72.3 (55.0 to 91.7)	77.5 (58.4 to 100)	0.686
Median CA difference, ° (IQR)	1.0 (−0.5 to 2.0)	−2.0 (−3.3 to −0.8)	0.019
CA difference within ±5°, n (%)	6/6 (100)	6/6 (100)	N/A
**CA 10° to 25°**			
Median accuracy, % (IQR)	90.5 (80.0 to 95.2)	90.9 (80.0 to 100)	0.837
Median CA difference, ° (IQR)	−1.0 (−3.0 to 1.0)	−1.0 (−2.0 to 0)	0.669
CA difference within ±5°, n (%)	19/19 (100.0)	17/19 (89.5)	0.146
**CA > 25° to 40°**			
Median accuracy, % (IQR)	92.6 (88.9 to 95.0)	86.2 (80.0 to 96.3)	0.146
Median CA difference, ° (IQR)	−1.0 (−3.0 to 2.0)	−1.0 (−6.0 to 4.0)	0.835
CA difference within ±5°, n (%)	15/15 (100)	8/15 (53.3)	0.003
**CA > 40°**			
Median accuracy, % (IQR)	96.3 (92.9 to 98.2)	81.5 (76.5 to 90.3)	<0.001
Median CA difference, ° (IQR)	0 (−3.0 to 2.0)	−9 (−12.5 to −4.8)	<0.001
CA difference within ±5°, n (%)	29/30 (96.7)	7/30 (23.3)	<0.001

Note: The chi-square test and the Mann-Whitney U test were performed for nominal and non-normally distributed variables, respectively. Cobb Angle (CA); Automated Cobb Angle Measurement Method (ACAMM); Interquartile Range (IQR).

**Table 6:** The reliability of the proposed ACAMM.

	ICC	PCC
Observer 1–Observer 2	0.957	0.948 (*p* < 0.001)
Observer 1–ACAMM	0.995	0.991 (*p* < 0.001)
Observer 2–ACAMM	0.954	0.939 (*p* < 0.001)

Note: Automated Cobb Angle Measurement Method (ACAMM); Intraclass Correlation Coefficient (ICC); Pearson Correlation Coefficient (PCC).
